# Assessment of Digital Pathology Imaging Biomarkers Associated with Breast Cancer Histologic Grade

**DOI:** 10.3390/curroncol28060366

**Published:** 2021-10-27

**Authors:** Andrew Lagree, Audrey Shiner, Marie Angeli Alera, Lauren Fleshner, Ethan Law, Brianna Law, Fang-I Lu, David Dodington, Sonal Gandhi, Elzbieta A. Slodkowska, Alex Shenfield, Katarzyna J. Jerzak, Ali Sadeghi-Naini, William T. Tran

**Affiliations:** 1 Department of Radiation Oncology, Sunnybrook Health Sciences Centre, Toronto, ON M4N 3M5, Canada; andrew.lagree@sri.utoronto.ca (A.L.); audrey.shiner@sri.utoronto.ca (A.S.); marieangeli.alera@sri.utoronto.ca (M.A.A.); lauren.fleshner@sri.utoronto.ca (L.F.); ethan.law1@sri.utoronto.ca (E.L.); brianna.law@sri.utoronto.ca (B.L.); asn@yorku.ca (A.S.-N.); 2 Biological Sciences Platform, Sunnybrook Research Institute, Toronto, ON M4N 3M5, Canada; 3 Temerty Centre for AI Research and Education, University of Toronto, Toronto, ON M5S 1A8, Canada; 4 Radiogenomics Laboratory, Sunnybrook Health Sciences Centre, Toronto, ON M4N 3M5, Canada; fangi.lu@sunnybrook.ca (F.-I.L.); Sonal.Gandhi@sunnybrook.ca (S.G.); 5 Department of Laboratory Medicine and Molecular Diagnostics, Sunnybrook Health Sciences Centre, Toronto, ON M4N 3M5, Canada; david.dodington@mail.utoronto.ca (D.D.); elzbieta.slodkowska@sunnybrook.ca (E.A.S.); 6 Division of Medical Oncology, Department of Medicine, University of Toronto, Toronto, ON M5S 3H2, Canada; katarzyna.jerzak@sunnybrook.ca; 7 Department of Engineering and Mathematics, Sheffield Hallam University, Howard St, Sheffield S1 1WB, UK; a.shenfield@shu.ac.uk; 8 Department of Electrical Engineering and Computer Science, York University, Toronto, ON M3J 2S5, Canada; 9 Department of Radiation Oncology, University of Toronto, Toronto, ON M5T 1P5, Canada

**Keywords:** breast cancer, Nottingham grade, tumor, biopsy, imaging biomarkers, computational oncology

## Abstract

Background: Evaluating histologic grade for breast cancer diagnosis is standard and associated with prognostic outcomes. Current challenges include the time required for manual microscopic evaluation and interobserver variability. This study proposes a computer-aided diagnostic (CAD) pipeline for grading tumors using artificial intelligence. Methods: There were 138 patients included in this retrospective study. Breast core biopsy slides were prepared using standard laboratory techniques, digitized, and pre-processed for analysis. Deep convolutional neural networks (CNNs) were developed to identify the regions of interest containing malignant cells and to segment tumor nuclei. Imaging-based features associated with spatial parameters were extracted from the segmented regions of interest (ROIs). Clinical datasets and pathologic biomarkers (estrogen receptor, progesterone receptor, and human epidermal growth factor 2) were collected from all study subjects. Pathologic, clinical, and imaging-based features were input into machine learning (ML) models to classify histologic grade, and model performances were tested against ground-truth labels at the patient-level. Classification performances were evaluated using receiver-operating characteristic (ROC) analysis. Results: Multiparametric feature sets, containing both clinical and imaging-based features, demonstrated high classification performance. Using imaging-derived markers alone, the classification performance demonstrated an area under the curve (AUC) of 0.745, while modeling these features with other pathologic biomarkers yielded an AUC of 0.836. Conclusion: These results demonstrate an association between tumor nuclear spatial features and tumor grade. If further validated, these systems may be implemented into pathology CADs and can assist pathologists to expeditiously grade tumors at the time of diagnosis and to help guide clinical decisions.

## 1. Introduction

Pathologic assessment is essential for breast cancer (BC) diagnosis and provides important histologic information to guide therapy. Standard specimen reporting guidelines from the College of American Pathologists (CAP; 2021) recommend biomarker analysis on diagnostic biopsies, including estrogen receptor (ER), progesterone receptor (PR), and human epidermal growth factor receptor-2 (HER2), for invasive breast carcinomas [1]. The CAP breast protocol also includes reporting histologic type, lymphovascular space involvement, and histologic grade as standard practice [1]. All of these parameters are important markers used to inform clinical decisions in breast oncology. Histologic grade was first introduced by Bloom and Richardson (1957), then modified by Elston and Ellis (1991), and is widely known today as the Nottingham grade [2]. The grading system is a semiquantitative method to assess morphological characteristics of tumor cells, specifically scoring tubule formation, nuclear pleomorphism, and mitotic activity. The combined score from these subcomponents results in classification among three grades (i.e., Nottingham grade 1–3), which signifies the level of differentiation from normal breast epithelial cells [2]. In terms of clinical utility, previous studies have shown an association to survival endpoints [3] and response to therapy [4], and recent guidelines from the American Joint Committee on Cancer (AJCC) have incorporated histologic grade into staging information, which is used, in part, to guide treatment strategies [5,6].

Manual annotations involve specimen preparation, sectioning, staining with hematoxylin and eosin (H&E), and evaluation under brightfield microscopy. Challenges associated with manual scoring approaches have been reported, including reproducibility issues and interobserver variability [2,7,8], with Kappa values reported between 0.43 to 0.85 [2,9,10]. However, one of the greatest challenges is the high demand on pathology resources, including the time required to evaluate cases. This is impacted by fluctuations in expertise and the need to manage other pathology tasks, such as administrative and operational functions [11,12]. To address these challenges, there is great interest in developing a stratification pipeline to identify and prioritize high-risk cases for expedited review [11,13]. Recent shifts toward high-resolution digital pathology imaging and the rapid growth in artificial intelligence (AI) in medicine have afforded exciting opportunities to achieve this by developing computational pathology, specifically computer-aided diagnostic (CAD) systems in pathology.

CAD systems have been developed in other medical specialties, including breast radiology, with the primary task of detecting and segmenting tumor masses on mammography [14]. In pathology, there are similar applications, i.e., AI-assisted tools to detect malignant regions on whole slide images (WSIs). Indeed, this has been the focus of several studies targeting lung [15], prostate [16,17], nasopharyngeal [18], and gynecological malignancies [19]. Developing CADs for breast cancer pathology is an area of clinical importance. In part, this is due to the biological complexity of breast tumors, and there is a need to initiate treatments early for high-risk disease, which depends on rapid diagnoses [20]. Automation to enhance efficiency and standardization to improve accuracy are important principles in this realm as well. Although CADs for breast pathology are undergoing development, several deep learning (DL) architectures for macroscopic region-based segmentation have been proposed using convolutional neural networks (CNNs) [21,22,23,24]. Similarly, DL architectures have been proposed for microscopic analysis of individual tumor cells and nuclei contained in the regions of interest (ROI), and this has the potential to yield richer information about tumor activity by using high-throughput computing to evaluate fine-grain tumor patterns and microscopic characteristics. Quantitating morphological and spatial attributes may provide insight into cell–cell interactions and characterize aggressive phenotypes, such as high histologic grade (i.e., Nottingham G3). Within this framework, CADs directed for microscopic analyses can fulfil three fundamental operations on diagnostic WSIs: (1) object recognition (e.g., cell and nuclear detection amidst the parenchymal background), (2) object classification (e.g., labeling tumor cells and nuclei from other cell types and stromal background), and (3) feature extraction (e.g., quantitative digital pathology imaging markers) [21,25]. The opportunities include validating features as markers for prognosis, treatment endpoints, and tumor phenotyping.

Several networks for microscopic object detection and segmentation have been proposed for breast biopsies [26,27,28,29]. Janowczyk et al., reported an efficient pipeline to segment breast tumor nuclei [28]. Their study included 137 breast cancer cases containing 141 regions for analysis using a so-called resolution adaptive deep hierarchical (RADHicaL) learning scheme [28]. The algorithm is predicated on a pixel-wise classification approach and includes a pre-processing step to rescale input images to increase computational efficiency. The DL backbone comprises an adapted AlexNet for classification [28]. The adaptive algorithm demonstrated good classification performances; the true-positive rate (TPR) and positive predictive value (PPV) were 0.8061 and 0.8822, respectively [28]. Other research has focused on automated immunohistochemistry (IHC) to evaluate biomarkers, such as HER2 [30]. Vandenberghe et al. compared two computational pipelines to analyze breast tumors, which used imaging features to carry out classification tasks. The first model consisted of a machine learning (ML) workflow, and performances were compared to a deep neural network. The CNN outperformed conventional ML models and showed an overall accuracy of 0.78 as well as demonstrated high concordance to pathologists’ assessments [30]. Overall, these studies demonstrate the ongoing interest to enhance automation for breast cancer diagnosis. In this present study, we build on our previous work [31,32,33] to develop CADs for pathology and propose a computational pipeline for histologic grading.

## 2. Methods

### 2.1. Patients and Dataset

**Figure 1:**
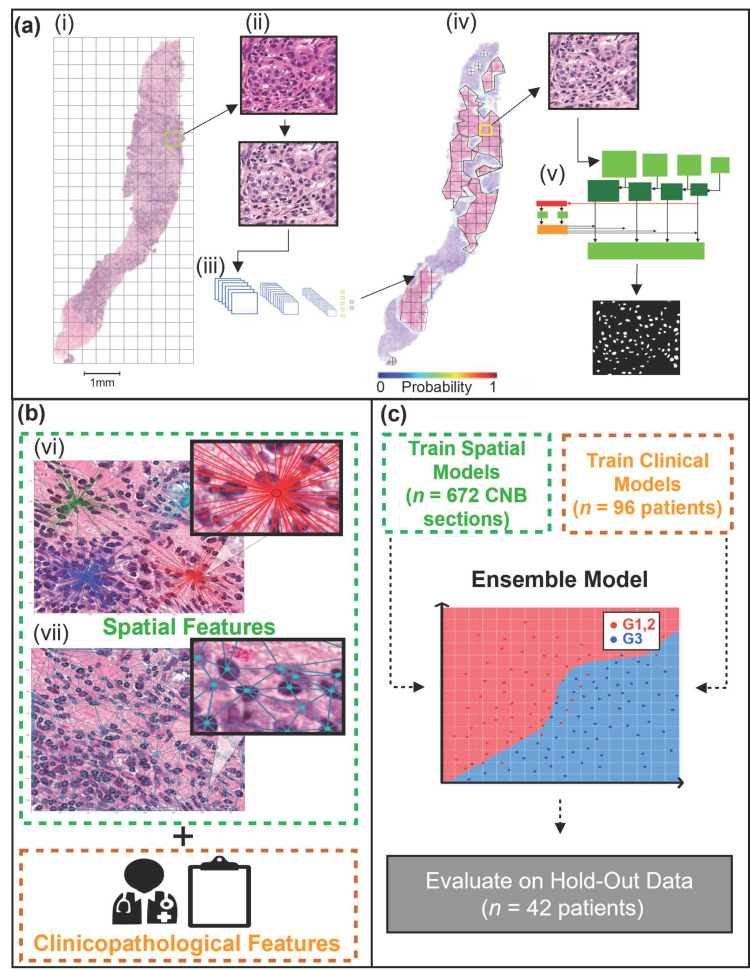
Nottingham grade classification pipeline. (**a**) (i) A representative H&E stained CNB section is first tiled, followed by stain normalization (ii), then used as input to a CNN (modified VGG19), which predicts the tumor bed probabilities (iii). A heatmap is generated using the tumor bed probabilities (iv). Tiles from the tumor bed are then used as input for the Mask R-CNN, which segments the malignant nuclei (v). (**b**) Spatial and clinical features are extracted. Spatial features were extracted using the centroids of the segmented nuclei. The spatial features included density features (vi), graph features (vii), and nuclei count. Clinicopathological features, including patient age (years) and receptor status (ER, PR, HER2). (**c**) Separate machine learning models were trained for spatial and clinical features. The clinical and spatial models were then combined to create an ensemble model. The ensemble model was evaluated on the hold-out (test) set.

A summary of the methods and analysis pipeline is presented in Figure 1. This study was a non-consecutive retrospective, single-institution study. All study parameters were approved by the institutional research ethics board prior to data collection and analysis. The study cohort consisted of biopsy-confirmed breast cancer patients who underwent anthracycline and taxane-based neoadjuvant chemotherapy (NAC) between 2013–2018 at Sunnybrook Health Sciences Centre (Toronto, ON, Canada). Patients were excluded from the study based on the following criteria: incomplete reporting of clinical-pathological data, metastatic disease presentation, incomplete course of NAC treatment, and administration of trial agents. Additionally, patients with invasive lobular carcinoma (ILC) were excluded from the study, as higher mitotic count of ILC correlates with higher stage and decreased survival; both nuclear pleomorphism and tumor architecture have not been shown to be prognostically significant [34,35]. Furthermore, their spatial organization demonstrates distinct patterns previously characterized as linear cellular arrangements, sheets, or nests [36].

Pathological reporting of Nottingham grade (G1, G2, G3) on pre-treatment core needle biopsies (CNB) was used as the ground truth labels for this study. As the primary aim of the study’s pipeline was to identify high-risk breast cancer (G3), the patients that presented with G1 or G2 were combined into a single class of low-intermediate grades. Tumor grade was reported by board-certified pathologists as part of the patient’s standard of care.

Other clinicopathologic data collection included patient age (years) and receptor status (ER, PR, HER2). ER, PR, and HER2 receptor status was assessed by immunohistochemistry (IHC); tumors with a HER2-equivocal score underwent dual-probe fluorescent and/or silver in-situ hybridization (FISH/SISH) to confirm the HER2 status. ER, PR, and HER2 status were defined using the American Society of Clinical Oncology (ASCO)/CAP guidelines [37,38,39]. All clinical and pathological data were extracted from the institution’s electronic medical record system. Other markers, such as Ki-67 immunohistochemistry, were not collected, as this was not part of the institution’s standard of care.

### 2.2. Specimen Preparation

CNBs were sectioned from formalin-fixed paraffin-embedded (FFPE) blocks, microtomed into 4 μm sections, and stained using H&E. Specimens were prepared onto glass slides for imaging. A commercially available digital pathology image scanner (TissueScope LE, Huron Digital Pathology Inc., St. Jacobs, ON, Canada) was used to digitize slides into WSI at high magnification (40×). Quality checks were performed on all WSI prior to analysis; all samples were verified for blurriness, staining irregularities, and external artifact contamination.

### 2.3. WSI Pre-Processing and Tumor Bed Identification

The first step in the digital analysis of the WSI was to separate the tissue (foreground) from the background. Tissue separation was accomplished by implementing Otsu thresholding and morphological operations (binary closing, removal) [40] to create a mask of each CNB section. Once masked, each section was separated from the remainder of the tissue on the WSI. The sections were then tiled (750 × 750 pixels), and each tile contained a maximum of 10% background (Figure 1a(i)). Furthermore, the tiles were stain normalized [41] (Figure 1a(ii)). A CNN was implemented to identify the tumor bed of each section (Figure 1a(iii)). The CNN, outlined in a previous study [33], took H&E input images of 750 × 750 pixels and returned a vector, which contained the probability of the tile belonging to the tumor bed. The probabilities were then used to re-build the original WSI, outlining the location of the tumor bed (Figure 1a(iv)). Once the tumor bed was identified, a tumor bed ratio (TBR) was calculated. The TBR of each CNB section was calculated by dividing the tumor bed area (pixels) by the sum of all tumor bed areas (pixels) within the WSI.

### 2.4. Instance Segmentation Network

Following tumor bed identification, the malignant nuclei within each tumor bed were segmented (Figure 1a(v)). To segment the nuclei a mask regional convolutional neural network (Mask R-CNN) was trained using the post-neo-adjuvant therapy breast cancer (Post-NAT-BRCA) dataset [42]. The dataset contained 37 WSI with 120 ROIs from breast resections of patients with residual invasive cancer following neoadjuvant therapy. Additionally, the dataset contained ground truth annotations performed by an expert pathologist of lymphocytes, normal epithelial, and malignant epithelial cells within each ROI.

For this study, the annotated ROIs were re-sized to a uniform dimension of 512 × 512 pixels. The images were then randomly partitioned into a training set (90%) and an independent test set (10%). The training images were further tiled to a dimension of 256 × 256 pixels. There were 480 training images in total, randomly split at a ratio of 80:20 between the training and validation datasets. Mask R-CNN was trained using residual networks (ResNet-101) [43] as the CNN backbone, initialized with weights pre-trained on the common objects in context (COCO) [44] dataset, and optimized with Stochastic Gradient Descent (SGD) [45]. A random image augmentation pipeline was implemented during training, which applied random combinations of flips, rotations, image scaling, and blurring to the images. Furthermore, SGD was set to a learning rate of 1 × 10^−4^, momentum of 0.9, and weight regularizer of 1 × 10^−4^. Mask R-CNN was trained until convergence, and the performance was evaluated on the validation set during training. Mask R-CNN was further evaluated using the unseen test dataset after training. Once trained on the Post-NAT-BRCA dataset, Mask R-CNN provided instance segmentation of the cells within the tumor bed for the 138 CNBs of the current study. Furthermore, only the malignant nuclei identified within the tumor bed were retained for analysis for this study.

### 2.5. Spatial Feature Extraction

Following nuclei segmentation, object-wise features were computed using the nuclear centroids; these included 52 spatial features per CNB section. There were three categories of spatial features adapted by methods previously described by Doyle et al. (2008) [46] and calculated using HistomicsTK [47]. Features were as follows: nuclear density features (Figure 1b(vi)), nuclear graph features (Figure 1b(vii)), and nuclear count. There were 24 nuclear graph features, encompassing Voronoi diagram features, Delaunay triangulation features, and Minimum Spanning Tree (MST) features. Furthermore, 27 nuclear density features were calculated, implementing *k*-dimensional (k–d) tree and ball tree algorithms. Lastly, the number of nuclei per CNB section was counted.

### 2.6. Machine Learning

Separate ML models were trained using clinical and spatial features. The test dataset was kept unseen during the development of each model, while the training dataset was used for feature reduction and model training (Figure 1c). First, a check of multi-collinearity was performed. A cross-correlation analysis was conducted, which identified highly correlated continuous features (r^2^ ≥ 0.7). The highly correlated features were then correlated with the outcome class (Nottingham grade) using point biserial; the feature with the highest correlation coefficient was retained. The data were then partitioned at the patient level into training (70%) and independent testing (30%) sets. The training data were standardized (Z-score normalization), and the means and standard deviations were retained to standardize the test set. To avoid class imbalance, the minority class (low-intermediate-grade BC [G1, 2]) was up-sampled using synthetic minority over-sampling technique (SMOTE) [48] and borderline SMOTE [49] for clinical and spatial features, respectively.

The following ML models were trained and evaluated: K-nearest neighbor (K-NN), logistic regression (LR), Naïve Bayes, support vector machines (SVM), random forest classifier (RF), and extreme gradient boost (XGBoost). As Naïve Bayes was not suitable for both continuous and ordinal features, it was excluded from clinical feature analysis. Sequential forward feature selection (SFFS) was performed with each ML model to identify the most discriminant features. The 10:1 rule for feature reduction was applied [50]; therefore, the maximum number of features permitted in the models was 10. SFFS was performed for 100 iterations per model. During each iteration, a 10-fold cross-validation (CV) technique was applied and evaluated using the area under the curve (AUC) of the receiver operating characteristics (ROC) curve. The features that maximized the AUC during each iteration were retained. The most discriminant features were identified as the most frequently occurring feature set throughout the 100 iterations. Lastly, each ML model’s hyperparameters were tuned using the randomized grid search (RGS) algorithm. RGS was performed for 100 iterations; a 10-fold CV technique was applied and evaluated using AUC during each iteration.

The final step was to develop an ensemble ML model, which would aggregate the predicted probabilities of the clinical and spatial feature ML models. The challenge of aggregating the predicted probabilities was addressed in two phases. First, as the spatial models’ predictions were made at the level of the CNB section, each probability was weighted based on the TBR and averaged per patient. Next, the weighting between clinical and spatial model predictions was addressed. A range of values beginning at zero and increasing linearly by 0.01 to one was implemented as weighted thresholds. A zero threshold weighted the clinical model’s predicted probabilities at 100%, while one weighted the spatial model’s predicted probabilities at 100%. A 10-fold CV strategy was implemented using the training dataset at each threshold to determine the optimal threshold. The threshold that maximized the AUC was then evaluated on the test set.

### 2.7. Software and Hardware

The software used for this study was written in Python programming language version 3.7.6 [51]. Using the Matterport package [52], Mask R-CNN was trained and implemented with Keras version 2.3.1 [53] and Tensorflow version 2.1.0 [54]. Global cell graph features were calculated using HistomicsTK version 1.0.5 [47], while nearest neighbor density estimations were calculated using AstroML version 0.4.1 [55]. Furthermore, MLxtend version 0.18.0 was used for SFFS [56]. Scikit learns version 0.24.1 [57] and XgBoost version 1.3.3 [58] were used for the remainder of the ML pipeline. All experiments were performed on a workstation equipped with an AMD (Advanced Micro Devices, Inc., Santa Clara, CA, USA) Ryzen Threadripper 1920X 12-Core Processor, 64GB of RAM, and a single NVIDIA (NVIDIA Corporation, Santa Clara, USA) GeForce RTX 2080 Ti graphics processing unit (GPU).

## 3. Results

### 3.1. Clinicopathological Characteristics

The study cohort contained 138 patients who presented with invasive ductal carcinoma (IDC), and a diagnostic core biopsy was collected from each subject for analysis. Of the 138 patients, four subjects (3%) had a G1 BC, 54 (39%) had G2, and 80 (58%) had G3 tumors. As the primary aim of this study was to develop a computational pipeline to classify high-risk BC cases (Nottingham G3), the low-intermediate grades (G1, 2) were grouped. There were 58 (42%) patients classified as G1, 2, and 80 (58%) patients exhibited G3 tumors.

The distributions of the clinicopathological features are outlined in Table 1. In univariate analysis, there were more ER-positive (*p* < 0.000) and PR-positive (*p* = 0.008) patients with low and intermediate grade (G1, 2) tumors compared to high grade (G3) tumors. Moreover, the distributions of the patients’ BC subtype, based on receptor status, with respect to the entire cohort, ML training set, and independent hold-out (testing) set are presented in Table S1. There were twenty patients (14%) whose subtype did not match that of the four reported subtypes. The distributions of BC subtypes based on receptor status ensured sufficient group representation within the training and testing sets since histological grade varies according to these subtypes [59]. The clinicopathological features included in ML modeling were age (years), ER (%), PR (%), and HER2 (+/−).

### 3.2. Mask R-CNN Segmentation

**Figure 2:**
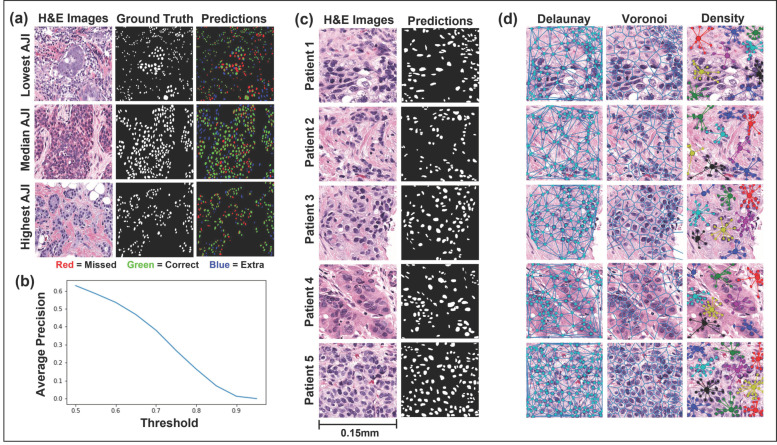
Instance segmentation of malignant nuclei by Mask regional convolutional neural network (Mask R-CNN) and representative feature extraction. (**a**,**b**) Mask R-CNN performance, evaluated on the hold-out (test) set. The highest, median, and lowest scoring AJI images from the Post-NAT-BRCA dataset are displayed. The predicted cells are color-coded such that green denotes true-positive, blue false-positive, and red false-negative pixels. Average precision over ten intersections over union thresholds is also displayed. (**c**) Representative H&E images from five patients and their respective malignant nuclei masks are displayed. (**d**) The Delaunay triangulation features, Voronoi diagram features, and density features were calculated using the centroids of the segmented malignant nuclei. Abbreviations: H&E, hematoxylin and eosin; AJI, Aggregated Jaccard Index.

Figure 2 displays the performance of the Mask R-CNN on the testing set (Figure 2a,b) and five representative H&E images from this study’s cohort (Figure 2c). Mask R-CNN achieved a mean Aggregated Jaccard Index (AJI) of 0.53 and a mean average precision (mAP) of 0.31. Furthermore, the network achieved F1, recall, and precision values of 0.65, 0.65, and 0.65, respectively, at an intersection over union (IoU) threshold of 0.5 and 0.40 at an IoU threshold of 0.7. Figure 2a displays the H&E images, ground truth annotations, and color-coded predictions for the lowest, median, and highest-scoring AJI images. The colors are coded such that green represents true-positive pixels, red represents false-negatives pixels, and blue represents false-positives pixels. In a qualitative review of the images, the network tended to over-segment the median and lowest AJI images and often failed to correctly segment nuclei where the image displayed staining irregularities. However, the network performed well in segmenting well-defined nuclei.

### 3.3. Computationally Derived Spatial Features

**Figure 3:**
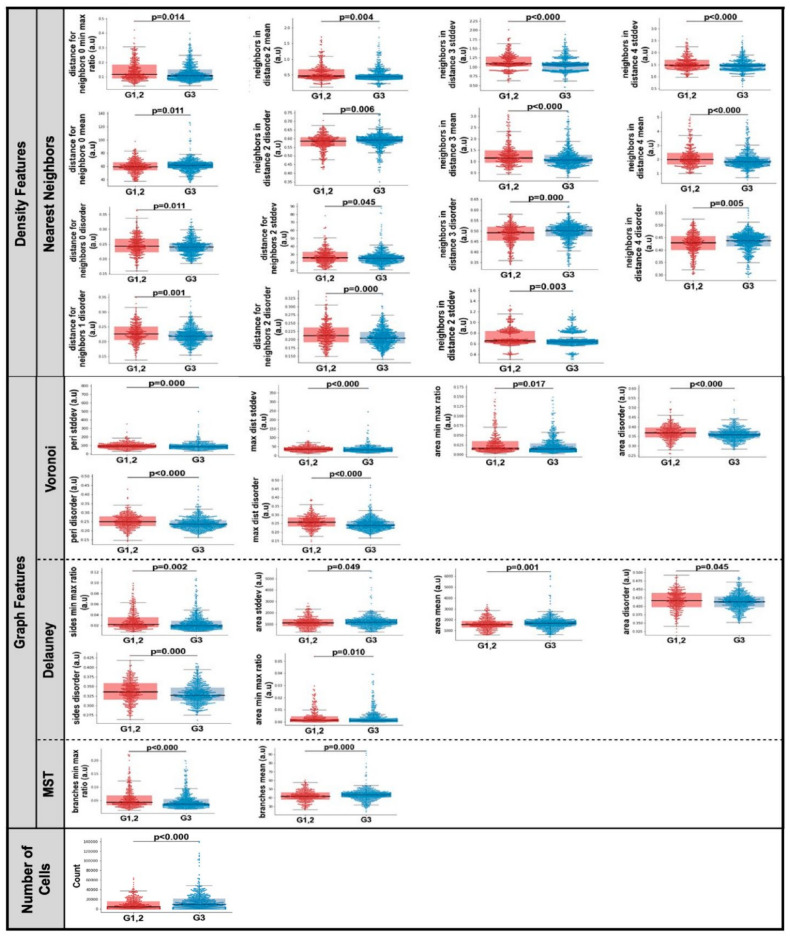
Combined box and whisker and swarm plots of the statistically significant (*p* < 0.05) spatial features. Abbreviations: stddev, standard deviation; Min, minimum; Max, maximum; MST, Minimum Spanning Tree; a.u., Arbitrary units; G1, 2, Nottingham grade 1 and 2; G3, Nottingham grade 3.

Fifty-two computationally derived nuclear spatial features were extracted from each CNB section. Representative features and group distributions are presented in Figure 2d. The centroids of the segmented nuclei were used to extract three categories of spatial features: nuclear density features, nuclear graph features, and nuclear count. In univariate analysis, thirty spatial features were significantly different (*p* < 0.05) in CNB sections of patients with G1, 2 compared to those with G3 (Figure 3). Of the thirty spatial features, fifteen included nearest neighbor density features, six were Voroni diagram features, six were Delaunay triangulation features, two were MST features, and significantly more nuclei were identified in CNB sections of patients with G3 tumors.

### 3.4. Predictive Modeling Using Machine Learning

Independent ML models were trained using spatial and clinical features. The ML models included Naïve Bayes, K-NN, LR, RF, SVM, and XGBoost; however, as Naïve Bayes is not suited for both continuous and ordinal features, it was excluded from clinical modeling. One hundred iterations of SFFS were performed to identify the most discriminant features and reduce the random effect of feature selection. A 10-fold CV strategy was implemented during each iteration, and the final feature sets were identified as the most frequently occurring features during the 100 iterations. Table 2 displays the most frequently occurring feature sets.

**Figure 4:**
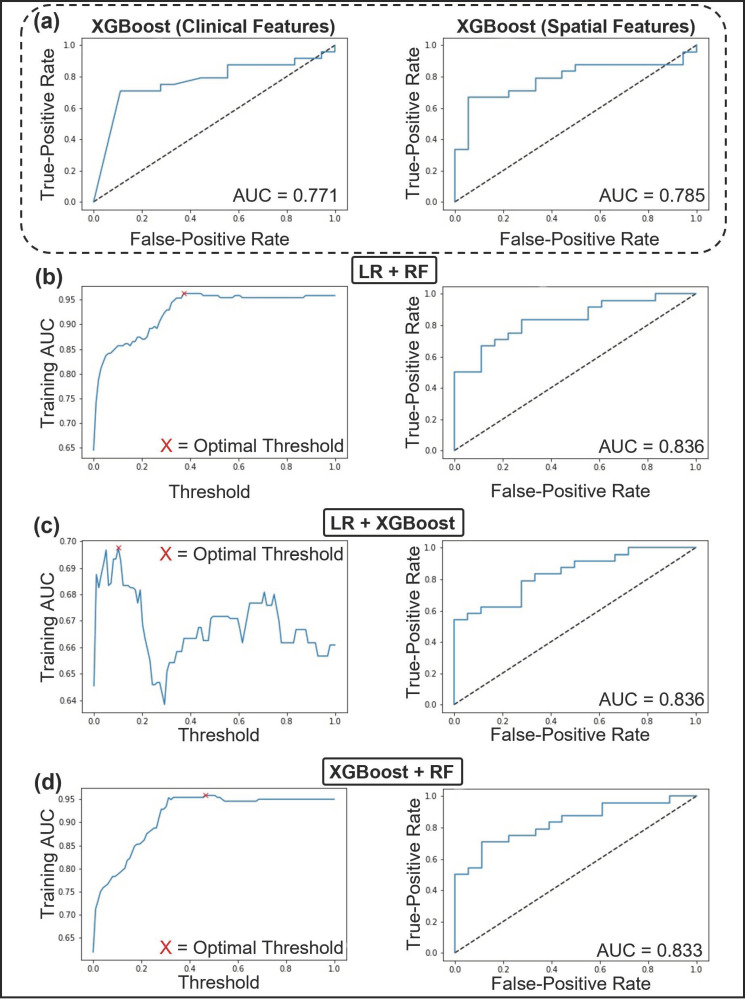
Receiver operating characteristics (ROC) curve and area under the curve (AUC) of the top performing machine learning models trained with clinical and spatial feature sets. (**a** left) ROC and AUC of XGBoost, the top performing classifier using clinical features. (**a** right) ROC and AUC of XGBoost, the top performing classifier using spatial features. (**b**–**d**) The top three performing ensemble models. (**b**) AUC vs. Threshold of LR+RF, with an optimal threshold of 37% (left). ROC and AUC of LR+RF (right). (**c**) AUC vs. Threshold of LR+XGBoost, with an optimal threshold of 10% (left). ROC and AUC of LR+XGBoost (right). (**d**) AUC vs. Threshold of XGBoost+RF, with an optimal threshold of 46% (left). ROC and AUC of XGBoost+RF (right). Abbreviations: ROC, Receiver Operating Characteristic curve; AUC, Area Under the Curve; LR, Logistic regression; RF, random forest classifier; XGBoost, Extreme Gradient Boost.

The RGS algorithm was used to tune each model’s hyperparameters. The hyperparameters that maximized AUC during 100 iterations of parameter selection are displayed in Table S2. The performance metrics, which represent the patient level Nottingham grade, of the clinical, spatial, and ensemble ML models are displayed in Table 3 and Table S3. Representative AUCs and weighting parameters are shown in Figure 4. The performance of the clinical ML models ranged from an AUC of 0.5–0.77 and 0.64–0.78 for the spatial models evaluated on the test set. The LR and XGBoost clinical models scored highest (AUC = 0.77, accuracy = 74%) on all metrics except for sensitivity and false-negative ratio, in which RF performed better. The best performing spatial model was XGBoost (AUC = 0.78, accuracy = 71%). All combinations of clinical and spatial ML models were evaluated in the development of the ensemble model. The ensemble model that performed best on the test set combined the LR clinical model and RF spatial model at a threshold of 0.37. The threshold weighting indicated that the clinical model predictions were weighted at 63%, while the spatial model predictions were weighted at 37%. The LR clinical model included ER (%) and PR (%) in the final analysis. The RF spatial model included the following features in the final analysis: nuclear count, Voronoi max distance standard deviation (SD), Voronoi max distance disorder, Density neighbors in distance one disorder, Density neighbors in distance 4 SD, Density distance for neighbors two minimum-maximum ratio, Density distance for neighbors two disorder, Density minimum, and Density median. The ensemble model achieved a mean AUC of 0.96 ± 0.12 and accuracy of 88 ± 14% during the 10-fold CV on the training data at a threshold of 0.37. The model further achieved an AUC of 0.84, accuracy of 78.57%, sensitivity of 83%, and specificity of 72% on the test set.

## 4. Discussion

In this study, we demonstrated that object-level (spatial) features derived from breast tumor WSI were associated with histologic grade. The results of our experiments showed good classification accuracy using machine learning to identify high Nottingham grade (G3) versus low- and intermediate-grade (G1,2) tumors. This study also demonstrated increased classification performances when ER/PR/HER2 pathologic biomarkers were included in the model.

In related works, previous approaches have been proposed for automatic histologic grading, including the use of various segmentation techniques, feature sets, group labeling conventions, and classification models [36,46,60,61,62,63,64]. Wan et al., examined 106 breast tumors and employed a hybrid active contour method to carry out segmentation tasks, using global and local image information [60]. Multi-level features were extracted, including texture (pixel-level), spatial (object-level), and semantic-level features derived from CNNs [60]. A SVM model was used for binary classifications (e.g., G1 versus G2,3), and another set of experiments were built on this pipeline to construct a cascade ensemble framework to classify G1 vs. G2 vs. G3. The results of their study showed an AUC of 0.87 ± 0.11 for binary classification of G3 from G1,2 tumors, and multivariate classification demonstrated an accuracy of 0.69 ± 0.12 for each Nottingham grade [60]. Other studies by Doyle et al., yielded an SVM classifier accuracy of 0.70 to distinguish low- versus high-grade tumors [46], and Cao et al., reported an accuracy of 0.90 by testing a combination of pixel-, object-, and semantic-level feature sets [61]. Recent research from Couture et al., implemented a DL-based segmentation step into their analysis pipeline for a large patient cohort (*n* = 579) [36]. Among histologic grade, other labels included ER status, histologic type, and molecular markers from their study population. Imaging data were yielded from digitized tissue microarray cores (TMA). The CNN backbone consisted of a VGG16 architecture for segmentation and feature extraction, followed by an ensemble SVM classifier. Nottingham labels were clustered into binary classes, which grouped patients as G1,2 versus G3. Ground truth labels were subjected to kappa statistics for interobserver agreement testing. With respect to histologic grade labels, applying a fixed threshold (0.8) showed an accuracy of 82% for detecting high-grade tumors [36]. Similarly, Yan et al., implemented an end-to-end computational pipeline, first using a deep learning framework for nuclear segmentation, then a Nuclei-Aware Network (NaNet) consisting of a VGG16 (Visual Geometry Group 16) backbone for feature representation learning [63]. Their results showed high classification accuracy (92.2%) from the model to distinguish each Nottingham category [63].

In comparison to these previous studies, our study aimed to focus on ML classification tasks based on object-wise spatial features and standard pathologic biomarkers; this was carried out by building an end-to-end ML pipeline consisting of a CNN-based segmentation framework, then computing imaging features from computed regions of interest. Finally, we exploited ML classification modeling to find features associated with the group labels. We compared and tested multiple classification models using these feature sets and found that the XGBoost model performed the best to automatically classify high-grade tumors; however, the ensemble models also performed well in correctly grading tumors. In designing this study, it was imperative to ensure sufficient representations within the high-grade class to train our model. Additionally, the group classes were determined based on its clinical relevance. Specifically, high-grade-tumors impact treatment and prognostic endpoints. Cortazar et al. demonstrated higher rates of pathologic complete response in high-grade tumors compared to those with grade 1/2 tumors for women treated with NAC [4]. Their pooled analysis also showed that exhibiting high-grade residual disease following NAC portended poorer survival outcomes [4]. This study also showed better classification performances by including standard biomarkers (ER/PR/HER2) in ML models. This may be explained by the association between high-grade tumors and aggressive subtypes, such as triple negative [65,66] and HER2-amplified [67] breast cancer.

Establishing robust and high-performance CAD systems in pathology has the potential to transform personalized medicine [68]. Indeed, pathologic evaluation is the gold-standard to derive a diagnosis and provides important information to steer treatment decisions in breast oncology, both in the neoadjuvant and adjuvant setting. Key opportunities for CADs in pathology include remote analysis and telepathology, which can bring expert review and automation to rural or underserviced regions. Additionally, computational pathology has the promise of advancing medical education curricula (e.g., provide learning materials and case series) and can facilitate quality initiatives in the laboratory and clinic as a second verification system to manual annotations [68,69]. Furthermore, CADs have the opportunity to assist with the reproducibility concerns of the current Nottingham grading guidelines [2]. Currently, manual microscopic review yields extensive variations in interobserver variability, with kappa values reported from fair to strong (0.43 to 0.85) [2,9,10]. Moreover, there is higher discordance in differentiating between G2 and G3 tumors and only fair interobserver agreement for classifying G2 tumors (K = 0.375) [9]. In the era of personalized oncology, digital biomarkers from pathology CADs may complement prediction and prognostic models and can be indexed into federated libraries to carry out population-based studies. Utilization of computational tools can also expedite the workflow, increase efficiency in the laboratory, and prioritize cases for review. Furthermore, it would provide a robust method of grading tumors, which would standardize the pathological workflow. Despite the array of opportunities, the challenges of digital pathology analysis include mechanical limitations of the imaging systems. There is a risk of suboptimal image quality (e.g., blurry images and software-generated artifacts) that have downstream effects on extracting features. Other considerations include determining optimal magnification and the use of multiplanar (cross sectional) views. Lastly, standardizing pre-processing methods is imperative to regulate staining intensities on variable samples as well as to mitigate the challenges of segmenting abutting or overlapping nuclei [70].

Limitations of this present study include a smaller patient cohort and the fact that models were trained and tested at a single institution. Future work will involve collection of data from annotated external data sets. As breast cancer represents an array of biological subtypes, another limitation includes the inability to generalize these findings with respect to BC intrinsic subtypes, e.g., luminal A, luminal B, triple negative, and HER2-amplified tumors. A larger cohort with sufficient samples will permit subtype analysis and aid feature learning and modeling. Another limitation of our study involves challenges with scoring intermediate-grade tumors, which have shown greater reporting variability among pathologists [9]. Thus, the grouping mechanism used in this present study (i.e., G1,2) could affect the classification performances of our model. Notwithstanding these limitations, this study demonstrates similar classification accuracy in comparison to previous studies and builds the framework for future work to include other biomarkers into the automatic annotation pipeline.

Prospective work may include refining computational frameworks across the pipeline to achieve optimal classification performances. Current efforts include automated assessment of tubule formation, nuclear pleomorphism, and mitotic activity. Adding spatial and pathologic feature sets to those experiments could potentially enhance the accuracy of grading tumors; this is achieved by adding meaningful features to the modeling dataset. Other areas of importance include training algorithms for ILC, as these tumors exhibit different morphological characteristics and spatial organization.

## 5. Conclusions

Developing CADs in pathology within the framework of AI and quantitative digital pathology imaging markers is poised to transform laboratory and clinical practices in oncology. Opportunities include case stratification, expedited review and annotation, and outputting meaningful models to guide treatment decisions and prognosticate patterns of breast cancer relapse and survival. To achieve this, robust and standardized computational, clinical, and laboratory practices need to be established in tandem and tested across multiple partnering sites for final validation. Nevertheless, with increased capacity in data informatics and processing, computational pathology will have an impact on breast cancer management.

## Figures and Tables

**Table 1:** Clinicopathological characteristics of the patients with G1, 2 and G3 breast cancer tumors. Bolded values represent statistical significance (*p* < 0.05). Abbreviations: G1, 2, Nottingham grade 1 and 2; G3, Nottingham grade 3; SD, standard deviation; y, years; ER, estrogen receptor; PR, progesterone receptor; HER2, human epidermal growth factor.

Patient Clinicopathological Characteristics	Study Cohort (*n* = 138)		
	G1, 2 (*n* = 58) *n* (%)	G3 (*n* = 80) *n* (%)	*p*-Value
Age			
Mean Age ± SD (y)	51.6 ± 10.8	50.3 ± 9.2	0.423
≤50 years	23 (39.7)	39 (48.8)	0.289
>50 years	35 (60.3)	41 (51.3)	
Menopausal Status			
Pre	30 (51.7)	38 (47.5)	0.624
Post	28 (48.3)	42 (52.5)	
Tumor Laterality			
Left	25 (43.1)	38 (47.5)	0.609
Right	33 (56.9)	42 (52.5)	
Receptor Status			
Median ER ± SD (%)	90 ± 44.1	0 ± 43.9	**<0.000**
ER-positive	43 (74.1)	34 (42.5)	**<0.000**
ER-negative	15 (25.9)	46 (57.5)	
Median PR ± SD (%)	4 ± 42.8	0 ± 36.3	**0.013**
PR-positive	35 (60.3)	30 (37.5)	**0.008**
PR-negative	23 (39.7)	50 (62.5)	
HER2-positive	26 (44.8)	39 (48.8)	0.649
HER2-negative	32 (55.2)	41 (51.3)	
Tumor Size			0.445
Mean Size ± SD (mm)	48.3 ± 27.8	44.5 ± 25.1	
Clinical T Stage			
1	5 (8.6)	4 (5.0)	0.314
2	32 (55.2)	54 (67.5)	
3	21 (36.2)	22 (27.5)	
4	0 (0.0)	0 (0.0)	
Clinical N Stage			
0	12 (20.7)	28 (35.0)	0.183
1	40 (69.0)	46 (57.5)	
2	6 (10.3)	6 (7.5)	
3	0 (0.0)	0 (0.0)	
Node Status			
Node-positive	46 (79.3)	52 (65.0)	0.067
Node-negative	12 (20.7)	28 (35.0)	
Inflammatory Breast Cancer			
Yes	5 (8.6)	8 (10.0)	0.784
No	53 (91.4)	72 (90.0)	

Bolded values represent statistical significance (*p* < 0.05).

**Table 2:** Most frequently occurring spatial and clinical feature sets. One hundred iterations of sequential forward feature selection were performed per model. The most frequently occurring clinical and spatial feature sets are reported. Abbreviations: K-NN, K-nearest neighbor; LR, logistic regression; RF, random forest classifier; SVM, support vector machine; XGBoost, extreme gradient boost; #, number; ρ, Density; V, Voronoi; D, Delauney; MST, Minimum Spanning Tree; Med, median; dist, distance; ƒ, frequency.

Model	Feature Type	Feature Index										
		1	2	3	4	5	6	7	8	9	10	ƒ
Naïve Bayes	Spatial	V max dist disorder	MST branches min max ratio									33
	Clinical											
K-NN	Spatial	# of nuclei	V max dist disorder	ρ neighbors in dist 1 disorder	ρ neighbors in dist 4 stddev	ρ min						16
	Clinical	Age (years)	ER (%)	PR (%)	HER2 status							63
LR	Spatial	V max dist stddev	V max dist disorder	MST branches min max ratio	ρ neighbors in dist 1 disorder	ρ dist for neighbors 2 min max ratio						43
	Clinical	ER (%)	PR (%)									81
RF	Spatial	# of nuclei	V max dist stddev	V max dist disorder	ρ neighbors in dist 1 disorder	ρ neighbors in dist 4 stddev	ρ dist for neighbors 2 min max ratio	ρ dist for neighbors 2 disorder	ρ min	ρ med		15
	Clinical	PR (%)	HER2 status									62
SVM	Spatial	# of nuclei	V max dist disorder	MST branches min max ratio	ρ neighbors in dist 1 disorder	ρ neighbors in dist 4 stddev	ρ dist for neighbors 2 disorder	ρ min	ρ med			25
	Clinical	Age (years)	ER (%)	PR (%)	HER2 status							84
XGBoost	Spatial	# of nuclei	V max dist stddev	V max dist disorder	MST branches min max ratio	ρ neighbors in dist 1 disorder	ρ neighbors in dist 4 stddev	ρ dist for neighbors 2 min max ratio	ρ dist for neighbors 2 disorder	ρ min	ρ med	7
	Clinical	ER (%)	PR (%)									22

**Table 3:** Performance measures of machine learning models, trained using clinical and spatial features sets. All models were trained using 10-fold cross-validation and tested on an independent hold-out set. The three highest performing ensemble models are reported. All performance measures are reported at the patient level. Abbreviations: K-NN, K-nearest neighbor; LR, logistic regression; RF, random forest classifier; SVM, support vector machine; XGBoost, extreme gradient boost; AUC, area under the curve; SD, standard deviation; ACC, accuracy; Sn, sensitivity; Sp, specificity; Prev, prevalence; FNR, false-negative rate; FPV, false-positive rate; PPV, positive predictive value; NPV, negative predictive value; FDR, false discovery rate; FOR, false omission rate; LR+, positive likelihood ratio; LR-, negative likelihood ratio; DOR, diagnostic odds ratio.

Feature Set	Model	Training Set		Testing Set														
		Mean AUC ± SD	Mean ACC ± SD (%)	AUC	Acc (%)	Sn (%)	Sp (%)	Prev (%)	FNR (%)	FPR (%)	PPV (%)	NPV (%)	FDR (%)	FOR (%)	LR+	LR-	DOR	f1
Clinical	K-NN	0.66 ± 0.16	62 ± 14	0.62	64.29	75.00	50.00	57.14	25.00	50.00	66.67	60.00	33.33	40.00	1.50	0.50	3.00	0.71
	LR	0.66 ± 0.22	64 ± 19	0.77	73.81	75.00	72.22	57.14	25.00	27.78	78.26	68.42	21.74	31.58	2.70	0.35	7.80	0.77
	RF	0.68 ± 0.15	59 ± 14	0.56	66.67	83.33	44.44	57.14	16.67	55.56	66.67	66.67	33.33	33.33	1.50	0.38	4.00	0.74
	SVM	0.64 ± 0.25	52 ± 16	0.50	66.67	75.00	55.56	57.14	25.00	44.44	69.23	62.50	30.77	37.50	1.69	0.45	3.75	0.72
	XGBoost	0.63 ± 0.23	59 ± 16	0.77	73.81	75.00	72.22	57.14	25.00	27.78	78.26	68.42	21.74	31.58	2.70	0.35	7.80	0.77
Spatial	Naïve Bayes	0.65 ± 0.07	59 ± 6	0.68	64.29	87.50	33.33	57.14	12.50	66.67	63.64	66.67	36.36	33.33	1.31	0.38	3.50	0.74
	K-NN	0.87 ± 0.03	76 ± 3	0.64	66.67	66.67	66.67	57.14	33.33	33.33	72.73	60.00	27.27	40.00	2.00	0.50	4.00	0.70
	LR	0.67 ± 0.04	62 ± 5	0.73	66.67	62.50	72.22	57.14	37.50	27.78	75.00	59.09	25.00	40.91	2.25	0.52	4.33	0.68
	RF	0.88 ± 0.04	79 ± 5	0.75	64.29	79.17	44.44	57.14	20.83	55.56	65.52	61.54	34.48	38.46	1.43	0.47	3.04	0.72
	SVM	0.79 ± 0.06	77 ± 5	0.69	69.05	75.00	61.11	57.14	25.00	38.89	72.00	64.71	28.00	35.29	1.93	0.41	4.71	0.73
	XGBoost	0.88 ± 0.03	79 ± 3	0.78	71.43	87.50	50.00	57.14	12.50	50.00	70.00	75.00	30.00	25.00	1.75	0.25	7.00	0.78
Ensemble	LR + RF	0.96 ± 0.12	88 ± 14	0.84	78.57	83.33	72.22	57.14	16.67	27.78	80.00	76.47	20.00	23.53	3.00	0.23	13.00	0.82
	LR + XGBoost	0.70 ± 0.23	56 ± 14	0.84	73.81	75.00	72.22	57.14	25.00	27.78	78.26	68.42	21.74	31.58	2.70	0.35	7.80	0.77
	XGBoost + RF	0.96 ± 0.13	92 ± 13	0.83	73.81	87.50	55.56	57.14	12.50	44.44	72.41	76.92	27.59	23.08	1.97	0.23	8.75	0.79

## Data Availability

The post-neo-adjuvant therapy breast cancer (Post-NAT-BRCA) dataset used in this study is available publicly at: https://wiki.cancerimagingarchive.net/pages/viewpage.action?pageId=52758117, accessed on 9 June 2020. The authors have made every effort to provide a detailed description of all data, software, and hardware used within this study. Data that have not been published alongside the article will be made available by the corresponding author upon reasonable request.

## Supplementary Materials

The following are available online at https://www.mdpi.com/article/10.3390/curroncol28060366/s1, Table S1: Relative proportions of the breast cancer subtypes, based on receptor status, with respect to the entire cohort, machine learning training, and testing sets, Table S2: Hyperparameters associated with the machine learning classifiers, Table S3: Performance measures of all ensemble models.

## References

[1] Fitzgibbons P.L., Connolly J.L. Cancer Protocol Templates. https://www.cap.org/protocols-and-guidelines/cancer-reporting-tools/cancer-protocol-templates.

[2] Van Dooijeweert C., van Diest P.J., Ellis I.O. (2021). Grading of invasive breast carcinoma: The way forward. Virchows Arch..

[3] Rakha E.A., El-Sayed M.E., Lee A.H.S., Elston C.W., Grainge M.J., Hodi Z., Blamey R.W., Ellis I.O. (2008). Prognostic significance of nottingham histologic grade in invasive breast carcinoma. J. Clin. Oncol..

[4] Cortazar P., Zhang L., Untch M., Mehta K., Costantino J.P., Wolmark N., Bonnefoi H., Cameron D., Gianni L., Valagussa P. (2014). Pathological complete response and long-term clinical benefit in breast cancer: The CTNeoBC pooled analysis. Lancet.

[5] Kalli S., Semine A., Cohen S., Naber S.P., Makim S.S., Bahl M. (2018). American Joint Committee on Cancer’s Staging System for Breast Cancer, Eighth Edition: What the Radiologist Needs to Know. RadioGraphics.

[6] Giuliano A.E., Edge S.B., Hortobagyi G.N. (2018). Eighth Edition of the AJCC Cancer Staging Manual: Breast Cancer. Ann. Surg. Oncol..

[7] Van Dooijeweert C., van Diest P.J., Baas I.O., van der Wall E., Deckers I.A. (2020). Variation in breast cancer grading: The effect of creating awareness through laboratory-specific and pathologist-specific feedback reports in 16,734 patients with breast cancer. J. Clin. Pathol..

[8] Dooijeweert C., Diest P.J., Willems S.M., Kuijpers C.C.H.J., Wall E., Overbeek L.I.H., Deckers I.A.G. (2020). Significant inter- and intra-laboratory variation in grading of invasive breast cancer: A nationwide study of 33,043 patients in The Netherlands. Int. J. Cancer.

[9] Ginter P.S., Idress R., D’Alfonso T.M., Fineberg S., Jaffer S., Sattar A.K., Chagpar A., Wilson P., Harigopal M. (2021). Histologic grading of breast carcinoma: A multi-institution study of interobserver variation using virtual microscopy. Mod. Pathol..

[10] Meyer J.S., Alvarez C., Milikowski C., Olson N., Russo I., Russo J., Glass A., Zehnbauer B.A., Lister K., Parwaresch R. (2005). Breast carcinoma malignancy grading by Bloom-Richardson system vs proliferation index: Reproducibility of grade and advantages of proliferation index. Mod. Pathol..

[11] Anglade F., Milner D.A., Brock J.E. (2020). Can pathology diagnostic services for cancer be stratified and serve global health?. Cancer.

[12] Metter D.M., Colgan T.J., Leung S.T., Timmons C.F., Park J.Y. (2019). Trends in the US and Canadian Pathologist Workforces From 2007 to 2017. JAMA Netw. Open.

[13] Krithiga R., Geetha P. (2021). Breast Cancer Detection, Segmentation and Classification on Histopathology Images Analysis: A Systematic Review. Arch. Comput. Methods Eng..

[14] Tran W.T., Sadeghi-Naini A., Lu F.-I., Gandhi S., Meti N., Brackstone M., Rakovitch E., Curpen B. (2021). Computational Radiology in Breast Cancer Screening and Diagnosis Using Artificial Intelligence. Can. Assoc. Radiol. J..

[15] AlZubaidi A.K., Sideseq F.B., Faeq A., Basil M. Computer aided diagnosis in digital pathology application: Review and perspective approach in lung cancer classification. Proceedings of the 2017 Annual Conference on New Trends in Information & Communications Technology Applications (NTICT).

[16] Chen C., Huang Y., Fang P., Liang C., Chang R. (2020). A computer-aided diagnosis system for differentiation and delineation of malignant regions on whole-slide prostate histopathology image using spatial statistics and multidimensional DenseNet. Med. Phys..

[17] Duran-Lopez L., Dominguez-Morales J.P., Conde-Martin A.F., Vicente-Diaz S., Linares-Barranco A. (2020). PROMETEO: A CNN-Based Computer-Aided Diagnosis System for WSI Prostate Cancer Detection. IEEE Access.

[18] Diao S., Hou J., Yu H., Zhao X., Sun Y., Lambo R.L., Xie Y., Liu L., Qin W., Luo W. (2020). Computer-Aided Pathologic Diagnosis of Nasopharyngeal Carcinoma Based on Deep Learning. Am. J. Pathol..

[19] Sun H., Zeng X., Xu T., Peng G., Ma Y. (2020). Computer-Aided Diagnosis in Histopathological Images of the Endometrium Using a Convolutional Neural Network and Attention Mechanisms. IEEE J. Biomed. Health Inform..

[20] Turashvili G., Brogi E. (2017). Tumor Heterogeneity in Breast Cancer. Front. Med..

[21] Hsu W.-W., Wu Y., Hao C., Hou Y.-L., Gao X., Shao Y., Zhang X., He T., Tai Y. (2021). A Computer-Aided Diagnosis System for Breast Pathology: A Deep Learning Approach with Model Interpretability from Pathological Perspective. arXiv.

[22] Fauzi M.F.A., Jamaluddin M.F., Lee J.T.H., Teoh K.H., Looi L.M. Tumor Region Localization in H&E Breast Carcinoma Images Using Deep Convolutional Neural Network. Proceedings of the 2018 Institute of Electronics and Electronics Engineers International Conference on Image Processing.

[23] Araújo T., Aresta G., Castro E., Rouco J., Aguiar P., Eloy C., Polónia A., Campilho A. (2017). Classification of breast cancer histology images using Convolutional Neural Networks. PLoS ONE.

[24] Zhang J., Guo X., Wang B., Cui W. (2021). Automatic Detection of Invasive Ductal Carcinoma Based on the Fusion of Multi-Scale Residual Convolutional Neural Network and SVM. IEEE Access.

[25] Chen J.-M., Li Y., Xu J., Gong L., Wang L.-W., Liu W.-L., Liu J. (2017). Computer-aided prognosis on breast cancer with hematoxylin and eosin histopathology images: A review. Tumor Biol..

[26] Xu J., Xiang L., Liu Q., Gilmore H., Wu J., Tang J., Madabhushi A. (2016). Stacked Sparse Autoencoder (SSAE) for Nuclei Detection on Breast Cancer Histopathology Images. IEEE Trans. Med. Imaging.

[27] Cireşan D.C., Giusti A., Gambardella L.M., Schmidhuber J. (2013). Mitosis Detection in Breast Cancer Histology Images with Deep Neural Networks. International Conference on Medical Image Computing and Computer-Assisted Intervention.

[28] Janowczyk A., Doyle S., Gilmore H., Madabhushi A. (2018). A resolution adaptive deep hierarchical (RADHicaL) learning scheme applied to nuclear segmentation of digital pathology images. Comput. Methods Biomech. Biomed. Eng. Imaging Vis..

[29] Veta M., van Diest P.J., Kornegoor R., Huisman A., Viergever M.A., Pluim J.P.W. (2013). Automatic Nuclei Segmentation in H&E Stained Breast Cancer Histopathology Images. PLoS ONE.

[30] Vandenberghe M.E., Scott M.L.J., Scorer P.W., Söderberg M., Balcerzak D., Barker C. (2017). Relevance of deep learning to facilitate the diagnosis of HER2 status in breast cancer. Sci. Rep..

[31] Lagree A., Mohebpour M., Meti N., Saednia K., Lu F.-I., Slodkowska E., Gandhi S., Rakovitch E., Shenfield A., Sadeghi-Naini A. (2021). A review and comparison of breast tumor cell nuclei segmentation performances using deep convolutional neural networks. Sci. Rep..

[32] Meti N., Saednia K., Lagree A., Tabbarah S., Mohebpour M., Kiss A., Lu F.-I., Slodkowska E., Gandhi S., Jerzak K.J. (2021). Machine Learning Frameworks to Predict Neoadjuvant Chemotherapy Response in Breast Cancer Using Clinical and Pathological Features. JCO Clin. Cancer Inform..

[33] Dodington D.W., Lagree A., Tabbarah S., Mohebpour M., Sadeghi-Naini A., Tran W.T., Lu F.-I. (2021). Analysis of tumor nuclear features using artificial intelligence to predict response to neoadjuvant chemotherapy in high-risk breast cancer patients. Breast Cancer Res. Treat..

[34] Rakha E.A., Van Deurzen C.H.M., Paish E.C., MacMillan R.D., Ellis I.O., Lee A.H.S. (2013). Pleomorphic lobular carcinoma of the breast: Is it a prognostically significant pathological subtype independent of histological grade?. Mod. Pathol..

[35] Bane A.L., Tjan S., Parkes R.K., Andrulis I., O’Malley F.P. (2005). Invasive lobular carcinoma: To grade or not to grade. Mod. Pathol..

[36] Couture H.D., Williams L.A., Geradts J., Nyante S.J., Butler E.N., Marron J.S., Perou C.M., Troester M.A., Niethammer M. (2018). Image analysis with deep learning to predict breast cancer grade, ER status, histologic subtype, and intrinsic subtype. NPJ Breast Cancer.

[37] Allison K.H., Hammond M.E.H., Dowsett M., McKernin S.E., Carey L.A., Fitzgibbons P.L., Hayes D.F., Lakhani S.R., Chavez-MacGregor M., Perlmutter J. (2020). Estrogen and progesterone receptor testing in breast cancer: American society of clinical oncology/college of American pathologists guideline update. Arch. Pathol. Lab. Med..

[38] Wolff A.C., McShane L.M., Hammond M.E.H., Allison K.H., Fitzgibbons P., Press M.F., Harvey B.E., Mangu P.B., Bartlett J.M.S., Hanna W. (2018). Human epidermal growth factor receptor 2 testing in breast cancer: American Society of Clinical Oncology/College of American Pathologists Clinical Practice Guideline Focused Update. Arch. Pathol. Lab. Med..

[39] Wolff A.C., Hammond M.E.H., Hicks D.G., Dowsett M., McShane L.M., Allison K.H., Allred D.C., Bartlett J.M.S., Bilous M., Fitzgibbons P. (2013). Recommendations for human epidermal growth factor receptor 2 testing in breast. J. Clin. Oncol..

[40] Otsu N. (1979). A Threshold Selection Method from Gray-Level Histograms. IEEE Trans. Syst. Man. Cybern..

[41] Vahadane A., Peng T., Sethi A., Albarqouni S., Wang L., Baust M., Steiger K., Schlitter A.M., Esposito I., Navab N. (2016). Structure-Preserving Color Normalization and Sparse Stain Separation for Histological Images. IEEE Trans. Med. Imaging.

[42] Martel A.L., Nofech-Mozes S., Salama S., Akbar S., Peikari M. Assessment of Residual Breast Cancer Cellularity after Neoadjuvant Chemotherapy Using Digital Pathology. https://wiki.cancerimagingarchive.net/pages/viewpage.action?pageId=52758117.

[43] He K., Zhang X., Ren S., Sun J. Deep Residual Learning for Image Recognition. Proceedings of the 2016 IEEE Conference on Computer Vision and Pattern Recognition (CVPR).

[44] Lin T.-Y., Maire M., Belongie S., Bourdev L., Girshick R., Hays J., Perona P., Ramanan D., Zitnick C.L., Dollár P. Microsoft COCO: Common Objects in Context. Proceedings of the IEEE Conference on Computer Vision and Pattern Recognition.

[45] Cauchy M.A. (1847). Méthode générale pour la résolution des systèmes d’équations simultanées. Comp. Rend. Hebd. Seances Acad. Sci..

[46] Doyle S., Agner S., Madabhushi A., Feldman M., Tomaszewski J. Automated grading of breast cancer histopathology using spectral clusteringwith textural and architectural image features. Proceedings of the 2008 5th IEEE International Symposium on Biomedical Imaging: From Nano to Macro.

[47] Gutman D.A., Khalilia M., Lee S., Nalisnik M., Mullen Z., Beezley J., Chittajallu D.R., Manthey D., Cooper L.A.D. (2017). The digital slide archive: A software platform for management, integration, and analysis of histology for cancer research. Cancer Res..

[48] Chawla N.V., Bowyer K.W., Hall L.O., Kegelmeyer W.P. (2002). SMOTE: Synthetic Minority Over-sampling Technique Nitesh. J. Artif. Intell. Res..

[49] Han H., Wang W.Y., Mao B.H. Borderline-SMOTE: A new over-sampling method in imbalanced data sets learning. Proceedings of the International Conference on Intelligent Computing.

[50] Harrell F.E., Lee K.L., Califf R.M., Pryor D.B., Rosati R.A. (1984). Regression modelling strategies for improved prognostic prediction. Stat. Med..

[51] Anaconda Software Distribution (2016). Computer Software. Vers. 2-2.3.1. https://www.anaconda.com/products/individual.

[52] Matterport’s Mask-R CNN Computer Software. https://github.com/matterport/Mask_RCNN.com.

[53] Chollet F. Others Keras. http://keras.io.

[54] Abadi M., Agarwal A., Barham P., Brevdo E., Chen Z., Citro C., Corrado G.S., Davis A., Dean J., Devin M. (2016). TensorFlow: Large-Scale Machine Learning on Heterogeneous Distributed Systems. arXiv.

[55] Vanderplas J., Connolly A.J., Ivezic Z., Gray A. Introduction to astroML: Machine learning for astrophysics. Proceedings of the 2012 Conference on Intelligent Data Understanding, CIDU 2012.

[56] Raschka S. (2018). MLxtend: Providing machine learning and data science utilities and extensions to Python’s scientific computing stack. J. Open Source Softw..

[57] Pedregosa F., Varoquaux G., Gramfort A., Michel V., Thirion B., Grisel O., Blondel M., Prettenhofer P., Weiss R., Dubourg V. (2011). Scikit-learn: Machine Learning in Python Fabian. J. Mach. Learn. Res..

[58] Chen T., Guestrin C. XGBoost: A scalable tree boosting system. Proceedings of the ACM SIGKDD International Conference on Knowledge Discovery and Data Mining 2016.

[59] Qiu J., Xue X., Hu C., Xu H., Kou D., Li R., Li M. (2016). Comparison of clinicopathological features and prognosis in triple-negative and non-triple negative breast cancer. J. Cancer.

[60] Wan T., Cao J., Chen J., Qin Z. (2017). Automated grading of breast cancer histopathology using cascaded ensemble with combination of multi-level image features. Neurocomputing.

[61] Cao J., Qin Z., Jing J., Chen J., Wan T. An automatic breast cancer grading method in histopathological images based on pixel-, object-, and semantic-level features. Proceedings of the 2016 IEEE 13th International Symposium on Biomedical Imaging (ISBI).

[62] Li L., Pan X., Yang H., Liu Z., He Y., Li Z., Fan Y., Cao Z., Zhang L. (2020). Multi-task deep learning for fine-grained classification and grading in breast cancer histopathological images. Multimed. Tools Appl..

[63] Yan R., Li J., Rao X., Lv Z., Zheng C., Dou J., Wang X., Ren F., Zhang F. NANet: Nuclei-Aware Network for Grading of Breast Cancer in HE Stained Pathological Images. Proceedings of the 2020 IEEE International Conference on Bioinformatics and Biomedicine (BIBM).

[64] Dimitropoulos K., Barmpoutis P., Zioga C., Kamas A., Patsiaoura K., Grammalidis N. (2017). Grading of invasive breast carcinoma through Grassmannian VLAD encoding. PLoS ONE.

[65] Dent R., Trudeau M., Pritchard K.I., Hanna W.M., Kahn H.K., Sawka C.A., Lickley L.A., Rawlinson E., Sun P., Narod S.A. (2007). Triple-Negative Breast Cancer: Clinical Features and Patterns of Recurrence. Clin. Cancer Res..

[66] Mills M.N., Yang G.Q., Oliver D.E., Liveringhouse C.L., Ahmed K.A., Orman A.G., Laronga C., Hoover S.J., Khakpour N., Costa R.L.B. (2018). Histologic heterogeneity of triple negative breast cancer: A National Cancer Centre Database analysis. Eur. J. Cancer.

[67] Lee H.J., Park I.A., Park S.Y., Seo A.N., Lim B., Chai Y., Song I.H., Kim N.E., Kim J.Y., Yu J.H. (2014). Two histopathologically different diseases: Hormone receptor-positive and hormone receptor-negative tumors in HER2-positive breast cancer. Breast Cancer Res. Treat..

[68] Acs B., Rantalainen M., Hartman J. (2020). Artificial intelligence as the next step towards precision pathology. J. Intern. Med..

[69] Nam S., Chong Y., Jung C.K., Kwak T.-Y., Lee J.Y., Park J., Rho M.J., Go H. (2020). Introduction to digital pathology and computer-aided pathology. J. Pathol. Transl. Med..

[70] Amitha H., Selvamani I. A Survey on Automatic Breast Cancer Grading of Histopathological Images. Proceedings of the 2018 International Conference on Control, Power, Communication and Computing Technologies (ICCPCCT).

