# Supplementary material for: Assessment of Digital Pathology Imaging Biomarkers Associated with Breast Cancer Histologic Grade
Source: Curr Oncol. 2021 Oct 27;28(6):4298–316. doi: 10.3390/curroncol28060366 (PMC8628688; doi:10.3390/curroncol28060366)
Supplement: Supplementary file 1 [file curroncol-28-00366-s001.zip › curroncol-1404716-supplementary.pdf]

Current Oncology

Supplementary Materials

Assessment of Digital Pathology Imaging Biomarkers Associated with Breast Cancer Histologic Grade

Andrew Lagree, Audrey Shiner, Marie Angeli Alera, Lauren Fleshner, Ethan Law, Brianna Law, Fang-I Lu, David Dodington, Sonal Gandhi, Elzbieta A. Slodkowska, Alex Shenfield, Katarzyna J. Jerzak, Ali Sadeghi-Naini and William T. Tran

Table S1. Relative proportions of the breast cancer subtypes, based on receptor status, with respect to the entire cohort, machine learning training, and testing sets. The subtype of 20 patients (14%) did not match that of the four reported subtypes. Abbreviations: ER, estrogen receptor; PR, progesterone receptor; HER2, Human Epidermal Growth Factor; TNBC, triple-negative breast cancer.

Subtype	–	Data Set	G1,2	G3	G1,2,3
			**n (%)**	**n (%)**	**n (%)**
ER+, PR+, HER2-		**Total**	**20 (14%)**	**13 (9%)**	**33 (24%)**
		Train	14 (10%)	8 (6%)	22 (16%)
		Test	6 (4%)	5 (4%)	11 (8%)
ER+, PR+, HER2+		**Total**	**13 (9%)**	**15 (11%)**	**28 (20%)**
		Train	10 (7%)	13 (9%)	23 (17%)
		Test	3 (2%)	2 (1%)	5 (4%)
ER-, PR-, HER2+		**Total**	**6 (4%)**	**18 (13%)**	**24 (17%)**
		Train	6 (4%)	11 (8%)	17 (12%)
		Test	0 (0%)	7 (5%)	7 (5%)
TNBC		**Total**	**7 (5%)**	**26 (19%)**	**33 (24%)**
		Train	5 (4%)	16 (12%)	21 (15%)
		Test	2 (1%)	10 (7%)	12 (9%)

Copyright: © 2021 by the authors. Licensee MDPI, Basel, Switzerland. This article is an open access article distributed under the terms and conditions of the Creative Commons Attribution (CC BY) license (http://creativecommons.org/licenses/by/4.0/).

Table 2. Hyperparameters associated with the machine learning classifiers. Hyperparameters were chosen using the randomized grid search algorithm for classifiers trained with clinical and spatial features. Abbreviations: SAGA, Stochastic Average Gradient variant; GB, gradient boost; RBF, radial basis function.

**Clinical Features**		**Spatial Features**	
		**K-Nearest Neighbour**	
**leaf size**	33	leaf size	31
neighbors	7	neighbors	5
		**Logistic Regression**	
C	0.3857	C	0.3857
penalty	L1	penalty	L1
solver	saga	solver	saga
		**Random Forest**	
maximum depth	16	maximum depth	20
minimum samples split	2	minimum samples split	2
number of estimators	246	number of estimators	377
		**Support Vector Machines**	
C	0.0003	C	138.4886
gamma	auto	gamma	scale
kernel	rbf	kernel	rbf
		**XGBoost**	
booster	gbtree	booster	gbtree
learning rate	0.01	learning rate	0.01
maximum depth	14	maximum depth	18
number of estimators	410	number of estimators	967
minimum child weight	3.3	minimum child weight	0.3

Table S3. Performance measures of all ensemble models. All ensemble models were trained using a 10-fold cross-validation technique at each threshold and tested on an independent hold-out set at the optimal threshold. All performance measures are reported at the patient level. Abbreviations: K-NN, K-nearest neighbour; LR, logisticreg ression; RF, random forest classifier; SVM, support vector machine; XGBoost, extreme gradient boost; AUC, area under the curve; SD, standard deviation; OT, optimal threshold; ACC, accuracy; Sn, sensitivity; Sp, specificity; Prev, prevalence; FNR, false-negative rate; FPV, false-positive rate; PPV, positive predictive value; NPV, negative predictive value; FDR, false discovery rate; FOR, false omission rate; LR+, positive likelihood ratio; LR-, negative likelihood ratio; DOR, diagnostic odds ratio.

**Feature Set**		**Training Set**		**Testing Set**															
**Clinical**	**Spatial**	**Mean AUC**	**Mean Acc (%)**	**OT (%)**	**AUC**	**Acc (%)**	**Sn (%)**	**Sp (%)**	**Prev (%)**	**FNR (%)**	**FPR (%)**	**PPV (%)**	**NPV (%)**	**FDR (%)**	**FOR (%)**	**LR+**	**LR-**	**DOR**	**f1**
K-NN	Naïve Bayes	0.74	58.22	91	0.68	64.29	87.5	33.33	57.14	12.5	66.67	63.64	66.67	36.36	33.33	1.31	0.38	3.5	0.74
	LR	0.75	60.22	90	0.73	64.29	62.5	66.67	57.14	37.5	33.33	71.43	57.14	28.57	42.86	1.88	0.56	3.33	0.67
	RF	0.96	93.89	59	0.72	64.29	70.83	55.56	57.14	29.17	44.44	68	58.82	32	41.18	1.59	0.53	3.04	0.69
	SVM	0.62	56	29	0.66	66.67	75	55.56	57.14	25	44.44	69.23	62.5	30.77	37.5	1.69	0.45	3.75	0.72
	XGBoost	0.7	61.11	72	0.77	71.43	83.33	55.56	57.14	16.67	44.44	71.43	71.43	28.57	28.57	1.88	0.3	6.25	0.77
LR	Naïve Bayes	0.73	59.33	61	0.8	66.67	83.33	44.44	57.14	16.67	55.56	66.67	66.67	33.33	33.33	1.5	0.38	4	0.74
	K-NN	0.7	55.89	3	0.78	73.81	75	72.22	57.14	25	27.78	78.26	68.42	21.74	31.58	2.7	0.35	7.8	0.77
	RF	0.96	87.67	37	0.84	78.57	83.33	72.22	57.14	16.67	27.78	80	76.47	20	23.53	3	0.23	13	0.82
	SVM	0.67	52.89	2	0.82	73.81	75	72.22	57.14	25	27.78	78.26	68.42	21.74	31.58	2.7	0.35	7.8	0.77
	XGBoost	0.7	55.89	10	0.84	73.81	75	72.22	57.14	25	27.78	78.26	68.42	21.74	31.58	2.7	0.35	7.8	0.77
RF	Naïve Bayes	0.84	70.89	54	0.63	66.67	83.33	44.44	57.14	16.67	55.56	66.67	66.67	33.33	33.33	1.5	0.38	4	0.74
	K-NN	0.87	77.11	25	0.6	69.05	79.17	55.56	57.14	20.83	44.44	70.37	66.67	29.63	33.33	1.78	0.38	4.75	0.75
	LR	0.85	68.67	66	0.67	71.43	75	66.67	57.14	25	33.33	75	66.67	25	33.33	2.25	0.38	6	0.75
	SVM	0.84	77.11	32	0.64	71.43	79.17	61.11	57.14	20.83	38.89	73.08	68.75	26.92	31.25	2.04	0.34	5.97	0.76
	XGBoost	0.87	73.89	39	0.66	69.05	79.17	55.56	57.14	20.83	44.44	70.37	66.67	29.63	33.33	1.78	0.38	4.75	0.75
SVM	Naïve Bayes	0.72	58.22	71	0.68	64.29	87.5	33.33	57.14	12.5	66.67	63.64	66.67	36.36	33.33	1.31	0.38	3.5	0.74
	K-NN	0.63	60.33	37	0.64	66.67	66.67	66.67	57.14	33.33	33.33	72.73	60	27.27	40	2	0.5	4	0.7
	LR	0.73	60.22	95	0.73	66.67	62.5	72.22	57.14	37.5	27.78	75	59.09	25	40.91	2.25	0.52	4.33	0.68
	RF	0.97	81	26	0.75	66.67	79.17	50	57.14	20.83	50	67.86	64.29	32.14	35.71	1.58	0.42	3.8	0.73
	XGBoost	0.67	57.11	46	0.78	71.43	87.5	50	57.14	12.5	50	70	75	30	25	1.75	0.25	7	0.78
XGBoost	Naïve Bayes	0.72	58.22	100	0.68	64.29	87.5	33.33	57.14	12.5	66.67	63.64	66.67	36.36	33.33	1.31	0.38	3.5	0.74
	K-NN	0.69	58.11	7	0.77	71.43	75	66.67	57.14	25	33.33	75	66.67	25	33.33	2.25	0.38	6	0.75
	LR	0.73	60.22	95	0.75	64.29	62.5	66.67	57.14	37.5	33.33	71.43	57.14	28.57	42.86	1.88	0.56	3.33	0.67
	RF	0.96	91.89	46	0.83	73.81	87.5	55.56	57.14	12.5	44.44	72.41	76.92	27.59	23.08	1.97	0.23	8.75	0.79
	SVM	0.65	56.22	13	0.82	69.05	70.83	66.67	57.14	29.17	33.33	73.91	63.16	26.09	36.84	2.13	0.44	4.86	0.72
